# The Case of Charles Sprague

**Published:** 1850-04

**Authors:** 


### ECLECTIC DEPARTMENT,

### AND SPIRIT OF THE MEDICAL PERIODICAL PRESS.

*The Case of Charles Sprague.*—Reported by C. H. Nichols, M. D., Physician to the Bloomingdale Asylum for the Insane, New York.

On the 10th of October, 1849, Charles Sprague was tried on an indictment for highway robbery, and acquitted on the ground of insanity; the trial taking place before a Court of Oyer and Terminer of King's County, N. Y., held in the City Hall of Brooklyn, Judge N. B. Morse, presiding.

Prosecuting Attorney, H. B. Duryea, Esq.; Counsel for defendant, John Dikeman and Alden Spooner, Esqs.

Deeming this case a clear one of irresponsibility on acccount of mental aberration, and one of at least as much psychological as forensic interest, it is not my purpose to give the particulars of the steps of this trial, but to make a suitable record of the history of a very singular case, of which its criminal relations are only an incident.

It may not be improper for me to state that I was present at this trial as an *expert*, and that I have derived all knowlege of the case from the testimony of the witnesses examined on the occasion under oath.

The principal witness was the defendant's father, a clergyman of the highest respectability, whose testimony was corroborated in every particular by several other witnesses, indeed by all the Court thought it worth while to have brought forward.

Charles Sprague's paternal great grandmother, grandmother, great uncle, and three great aunts, being four out of a family of six, and a cousin, are, or have been insane; a brother of his father is subject to fits of very excessive and apparently uncontrollable passion, and a sister of his has sudden paroxysms of intense groundless fear, and at such times is soothed with great difficulty.

When Charles was about seven years of age, he received upon his head a blow from a hoe, but it was not at the time supposed that the skull was injured; soon after this he fell from a height, bruising his head to some extent, and knocking out several of his teeth; and when past twelve he again fell from a balcony, striking in part upon his head, but the concussion was not at the time deemed alarming, for in the course of a few days after the accident, he went out to his school and play, and appeared as well as usual.

In the course of the year succeeding the last fall, however, he began to complain at times of pain in his head, and when suffering from headache, his friends observed what his father describes as an unnnatural prominence and dullness or glassiness of his eyes, and though the headache gradually wore away, this peculiarity in his expression continued to recur for years, at intervals varying from one week to several months, till it is now thought that there is a settled change in his eyes to greater prominence or convexity of the globe, than was the case in early boyhood. There has at no time been any unusual enlargement of the head or change in shape. Simultaneously or closely succeeding the first appearance of the occasional headache and strangeness in the expression of his eyes, there began to be developed a propensity of mind or occasional conduct, of its kind most extraordinary and unaccountable.

The *shoes* of the female members of the family were from time to time missing, under circumstances most surprising and inexplicable. It at once appeared that they could not have been taken for use, for, though both of a pair were sometimes lost, in the majority of instances one only was gone, and it was usually found about the house, having been thoroughly soaked in water, twisted up like a rope, and then hid away between the feather and straw bed, or in the depths of a trunk, or hung up in a clothes closet, with garments hung about it, on the same hook, as if to conceal it.

After much fruitless inquiry respecting the perpetrator of this mischief, a servant girl was accused of it, but she firmly denied it, and there the matter rested for a time. It was not long, however, before a shoe was missing under such circumstances, as to render it quite evident that Charles had taken it, and disposed of it in the manner before mentioned. When questioned upon the subject, he hung his head and was silent, and he again and again repeated the act, and was as often interrogated concerning it, but invariably with the like unsatisfactory result. After this mysterious habit had existed for a year or two, an attempt was made to break him of it by reproof of various kinds, and then he only broke silence in efforts to evade the subject, not to make any explanations regarding it. And at a later period, he would wholly deny the possibility of his having taken a shoe as alleged, but for the last half dozen years he has said when remonstrated with on account of his indulgence in a habit so singular and inconvenient, and the circumstance of the loss of a particular shoe, stated, that he *must* have taken it, though he had no recollection of the act, and did not know what he wanted with it.

This habit of taking the shoes of females commenced at the time stated above, and has continued up to the present moment, without, it is thought, any intermission of more than three or four months duration. After the practice became established, his mother and sisters, and the female servants in the family, made it a point to place all their valuable shoes under lock and key, but notwithstanding these precautions, he occasionally succeded in possessing himself of them, and when a shoe was missed, it was usually, sooner or later, discovered in some by-place about the house, having been wet and twisted or crumpled.

There was at one time a rumor in the family that this singularly affected son had attempted to remove a shoe from the foot of a servant girl in the house; and on one occasion, hearing a loud scream in the middle of the night, coming from the floor on which he and his sisters slept in contiguous bed-rooms, his father jumped from his bed, ran to his daughters' room and was informed by one of them that Charles had been in their room, and, without the aid of a light, turned the key of a drawer, taken out a pair of shoes which lay purposely concealed in a quantity of clothing, and then had come to the foot of her bed and pinched one of her toes. At this juncture she screamed, and her brother immediately dropped the shoes at the foot of the bed, and repaired to his own room, where his father found him in bed.

This propensity continued to manifest itself in the manner now described, till in the early part of the present year two females residing in the city had each a shoe or shoes taken off her feet, while walking in the street in the evening, but who the offender was in those cases was never known, nor is it now, but they are mentioned as being analagous to that about to be described, and it may be that one individual was concerned in them all.

Sometime in July last, Mr. C. Sprague's wife recollects that she purchased a pair of shoes for a particular occasion, and when she wanted them they had disappeared, and though it was a riddle to her, it became evident to his father, who was then boarding with him, that their disappearance was due to the exercise of his old propensity.

In August last, Mr. Sprague left his house immediately after breakfast, to go to his business, (that of a printer,) and in a few moments after was seen walking towards his house, instead of towards his office, for which he had set out, and to overtake a young lady, throw her down, snatch the shoe from one of her feet, and, on an outcry being made by several persons who were hard by, run away. The young lady wore a chain and locket and other jewelry in sight, but he did not offer to take any thing from her person but her shoe, nor did he do violence to her person in any respect or degree. Running, he proceeded round a square, and on his way called at his wife's father's, and asked if his father was in town, a matter upon which he was perfectly well informed, then left the house, came directly back to the very spot where he had just taken the shoe, and continued on, without stopping, to his place of business. He was soon arrested and taken before a magistrate, and when interrogated in regard to the shoe, he said he had changed his coat after going to the office, and that the shoe was in the pocket of the one he had taken off, where it was found. After the requisite process, he was committed to prison to answer the charge of highway robbery, and on the return to the city of his father, who was absent at the time of the unfortunate occurrence in the street, he was admitted to bail in the sum of $5000, and on the 19th of October his trial took place, as stated at the opening of this paper.

The relations between Mr. S. and his father had always been particularly genial, frank and confiding, and when they met the first time some days after the robbery, the former burst into tears, and the following dialogue took place, which I give in detail, as indicating the state of the young man's mind during the commission of the act in question:—

"Well, Charley, this is the same old thing?"

"Yes it is, father."

"What can you say about it?"

(Charles, looking into his father's face with an expression of perfect ingenuousness and honesty,) "Well, father, I don't know much of anything about it, except what they (those who witnessed the deed) told me."

"You don't deny that you did it, do you?"

"No, not at all."

"Tell me, my son, just what you remember about it."

"I think I was going along the street, and caught sight of a shoe, and it flashed into my mind like lightning that I wanted it, and I dove for it."

'But what did you want with that shoe?"

"I don't know what I did want with it."

"You know you have for years been in the habit of taking your mother and sister's shoes—what did you *ever* want with a shoe?"

"I don't know, father, what I ever did want with one."

"How did you get the girl down?"

"Don't know anything about it, only they told me I did get her down."

"Did you strike her or trip up her heels?"

"Don't know. *The whole affair is a kind of haze before my mind.* The first perfectly distinct recollection that I have of what took place that morning, is of being near the printing office after the affair with the young lady, about half a mile beyond where it occurred."

During the period which elapsed between Mr. S.'s arrest and final trial, his mind was at times so much agitated that his friends were not a little apprehensive of mental disease in its more common forms, and great pains were necessary to calm and soothe him, particularly in his anxiety respecting the issue of his approaching trial, and his old propensity was more active than for some time past. On one occasion his wife fell asleep while sitting in an easy chair, and when she awoke she perceived that one of the shoes she was then wearing, had been taken from her foot during her sleep. Making immediate search of the person of her husband, who was present, she found the missing shoe tucked into the leg of one of the boots that he then had on. At another time missing one of her shoes, she sent a messenger to the office where it was found in Mr. S.'s coat pocket.

Mr. Sprague's moral character has been singularly faultless, unless the propensity to take shoes be excepted. He has never been known to drink a glass of spirits, to use a profane word, or to keep vicious company. He has never been known to utter a falsehood in any other than a shoe case, or to take anything wrongfully except shoes. Nothing in his manner has ever afforded the least warning of the time of committing the acts inquestion, or betrayal of them after their commission. He has all his life resided either in his father's or brother's family, or kept house himself, and except the instance in the street, it is not certainly known that he has ever taken a shoe that did not belong to some member of the family of which he formed a part, and his friends are not aware that he has ever taken any but *women's* shoes. Of the hundreds of instances in which be has exercised this unique propensity, he has been *seen* to take a shoe only twice; once when he went to his sisters' lodging room at night, and took a pair of shoes hid in clothing contained in a locked bureau drawer, and again when he snatched a shoe from the foot of a young lady, in a broad open street, in the day-time and in the presence of many spectators.

As respects Mr. S.'s intellectual character:—Their father designed giving both his sons, this one and another a year or two older, a collegiate education, but it became quite apparent that study was neither attractive nor easy to Charles, though his inaptness for learning was by no means greater than is very common with minds whose integrity is never in the least suspected, the original design was abandoned, and, instead, after obtaining an education ample for business, he in accordance with his own wishes was bound as an apprentice to a printer. Pursuing his chosen calling uninterruptedly to the present time, first as an apprentice and then as higher journeyman, he has uniformly maintained a high character for integrity, regularity of habits and general efficiency. As the only drawback on this commendation, it is stated that when much hurried with irregular and unexpected jobs, he is apt to become confused and is unable to proceed until his mind becomes collected by a short relaxation. Knowing that he inherited a tendency to nervous derangements, after the development of a propensity so objectless in any rational point of view, his father apprehended that his mind was not well balanced, but aside from the one particular described, there has never been any mental manifestation, or any habit, that would of itself have raised the suspicion of insanity.

Mr. S. is most respectably married, and has one child.

The reader familiar with mental pathology will perceive that the case whose history is now given, is distinguished by several peculiarities not unusual in monomania,—the denomination of that class of diseases to which I have supposed this may best belong.

In respect to the one morbid propensity that has now been exhibited for upwards of ten years without material increase increase or diminution in intensity, or variation in general character, and without any supervening mental derangement, this case presents one of the very finest specimens of monomania on record. Monomania, however, is usually the morbid exercise of one or more of the normal faculties of the understanding. Unrestrained ambition after a time becomes diseased and in consequence emancipates itself from the control of reason, and then deems itself President or Emperor; diseased avarice steals; diseased revenge kills; and diseased religious sentiment certain of final condenmation, anticipates its doom by suicide; but in this case we have no idea where the disease begins—we cannot perceive a motive either rational or irrational, for there is no natural propensity to which this is analogous or eonsecutive. This is a propensity *sui generis*. A morbid propensity to steal, if it discriminates at all, usually does so in favor of articles that are useful, really or fanciedly.

In the unpremeditated suddenness of the wholly motiveless act in the street, there is a resemblance to those explosive manifestations of mental disorder that have received the appellation of "insane impulse."

In the avoidance of observation at the time of the commission of the act, and of allusion to it at other times, there is evidence of a degree of consciousness and design; but in the imperfect recollection of the act and its events, and of the state of the feelings at the time—in the "haze" that then clouds the mind—there is an analogy to somnambulism—that state in which there seems to be a suspension of self-consciousness, while the senses and other bodily powers are still exercised in obedience to the impulse of a waking imagination.—*Journal of Insanity.*

